# Multi-mode biodegradable tumour-microenvironment sensitive nanoparticles for targeted breast cancer imaging

**DOI:** 10.1186/s11671-020-03309-w

**Published:** 2020-04-15

**Authors:** Zhenhui Nie, Ningbin Luo, Junjie Liu, Xinyi Zeng, Yu Zhang, Danke Su

**Affiliations:** 1 Department of Radiology, Affiliated Tumour Hospital of Guangxi Medical University, No 71, Hedi Road, Nanning, 530021 Guangxi People’s Republic of China; 2 Department of Medical Ultrasound, Affiliated Tumour Hospital of Guangxi Medical University, No 71, Hedi Road, Nanning, 530021 Guangxi People’s Republic of China

**Keywords:** Ultrasound, Gas-generating nanoparticle, Multi-mode imaging, Tumour targeting, Contrast agent

## Abstract

Gas-filled ultrasound (US) contrast agents easily collapse in the body, and the gas can easily overflow, which limits the effectiveness of US imaging. To address this issue, an injectable gas-generating multi-mode system was developed that carries the MR negative contrast agent Fe_3_O_4_, the fluorescent dye Cy5.5, and the CO_2_ releasing donor (Na_2_CO_3_). The nanoparticles can continuously generate carbon dioxide (CO_2_) gas in acidic tumour tissue in the body, giving the tumour a strong echo signal under ultrasonic imaging. In addition, the nanoparticles confer excellent effects for MR and fluorescence imaging of the tumour tissue. The results indicate that this pH-responsive NP system provides good effects in MR/US/fluorescent imaging. This study provides a useful reference for multi-mode tumour imaging.

## Introduction

In clinical practice, microbubbles are primarily applied as ultrasound contrast agents for the real-time imaging of various organs and blood vessels [1–3]. Traditional ultrasound contrast agents typically consist of materials such as lipids or proteins that enclose air or perfluorocarbon gases. The gas species encapsulated in the microspheres have low stability in the blood and a short half-life because of the rapid diffusion of microbubble defects [4–6]. Moreover, because the particle size of the gas-filled microbubbles is usually large (approximately 1 to 8 μm), it is difficult for the microbubbles to penetrate into the host tumour environment by tissue extravasation. Therefore, the current application of micron-sized bubbles in intravascular imaging is limited [7]. Ideal ultrasound contrast agents should generally exhibit an optimal size for transport through the tissue vascular space, an adequate duration of acoustic effect, good targeting and biocompatibility, and easy excretion from the body [8, 9]. The concept of “gas-generating nanoparticles” was proposed in prior research, and such nanoparticles have the potential for use in ultrasound contrast imaging [10–12]. These gas-generating nanoparticles are superior to the current gas-filled microbubbles in performance, and the continuously generated gas enables intense ultrasound imaging. Gas-generating nanoparticles can enhance permeation and retention, and they may stably circulate in the blood and effectively accumulate in the tumour tissue [13, 14].

It remains a challenge to detect tiny and occult tumours by traditional imaging methods, such as magnetic resonance imaging (MRI), computed tomography (CT), and ultrasound, which are limited by long acquisition times, high radiation dose, and poor sensitivity [15, 16]. It is necessary to integrate different imaging methods and develop multi-modal imaging technology to achieve integrate synergy for the early detection of cancer [17–19]. Superparamagnetic iron oxide (Fe_3_O_4_) nanoparticles can be used as negative MRI contrast agents in T2-weighted imaging [20, 21]. Fe_3_O_4_ has attractive overall properties, including small particle size, strong penetrability, high magnetization, good metabolism, and relatively low toxicity [22, 23]. Fe_3_O_4_ contrast agents for the MRI diagnosis of early-stage cancer have been extensively studied due to their high relaxation and contrast [24–26]. In addition, real-time fluorescence imaging has excellent resolution and can be a valuable method for defining tumour staging, guiding tumour resection, and monitoring the treatment effects [27, 28].

**Scheme 1:**
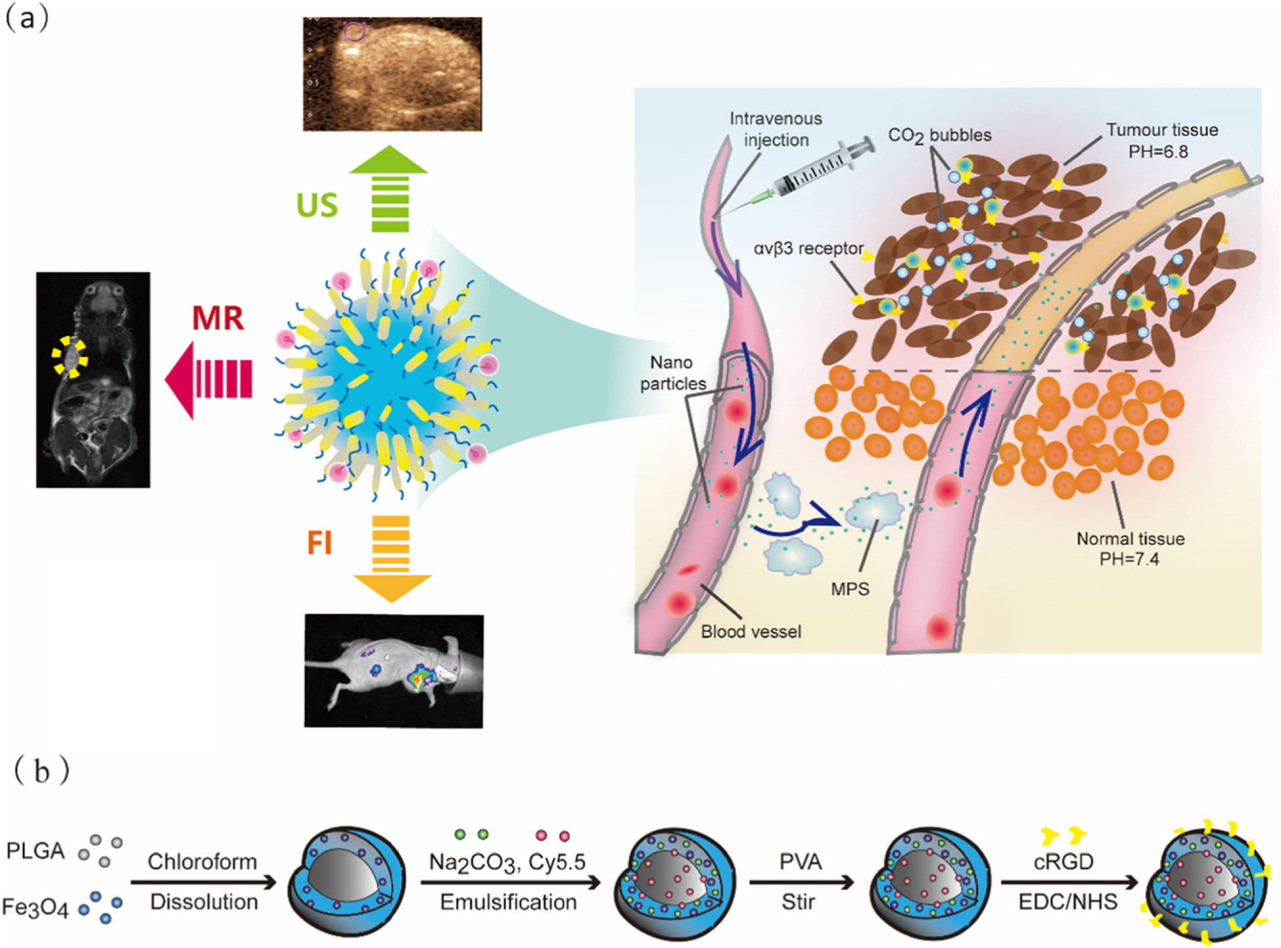
**a** Schematic diagrams of the function in Fe_3_O_4_/Na_2_CO_3_ @PLGA/Cy5.5/cRGD NPs via targeted accumulation into the tumor and generating CO_2_ bubbles in acid tumor tissues, followed by triple-modal MR/US/FI imaging of breast cancer. **b** Schematic illustration of the preparation of the Fe_3_O_4_/ Na_2_CO_3_ @PLGA/Cy5.5/cRGD NPs.

Herein, these nanoparticles are mainly encapsulated by poly (lactic-co-glycolic) acid (PLGA), which has been approved by the Food and Drug Administration (FDA) for use as a biosafe material [29, 30]. The PLGA particles are modified with RGD peptide to enable binding to αvβ3 integrin on the surface of breast cancer cells and with Cy5.5 as a fluorescent dye for imaging in vivo, and they are encapsulated with Fe_3_O_4_ to act as a T2-negative contrast agent in MRI (Scheme 1a). Due to the upregulated glycolysis in tumour tissue, which could produce more lactic acid and protons in the extracellular environment, the pH of tumour tissues (6.8–7.2) is lower than that of normal tissues (pH 7.4) [31–33]. Thus, we designed sodium carbonate (Na_2_CO_3_) in the PLGA to produce CO_2_ bubbles at the lower pH of tumour tissues for ultrasound imaging. To verify their promising application in tumour imaging, the comprehensive properties of these multi-mode nanoparticles for in vitro imaging were systematically characterized, including their cytotoxicity, targeting specificity, and biodistribution in tumour tissue, by three imaging modes.

## Results and discussion

### Synthesis and characterization of Na_2_CO_3_/Fe_3_O_4_ @PLGA/Cy5.5/RGD NPs

Na_2_CO_3_/Fe_3_O_4_@PLGA/Cy5.5/RGD NPs were designed as RGD-targeted multi-mode contrast agents by encapsulating a biocompatible polymer of PLGA with Fe_3_O_4_ and Na_2_CO_3_ and an integrin-targeted agent via a biodegradable chemical bond (Scheme 1b).

**Fig. 1:**
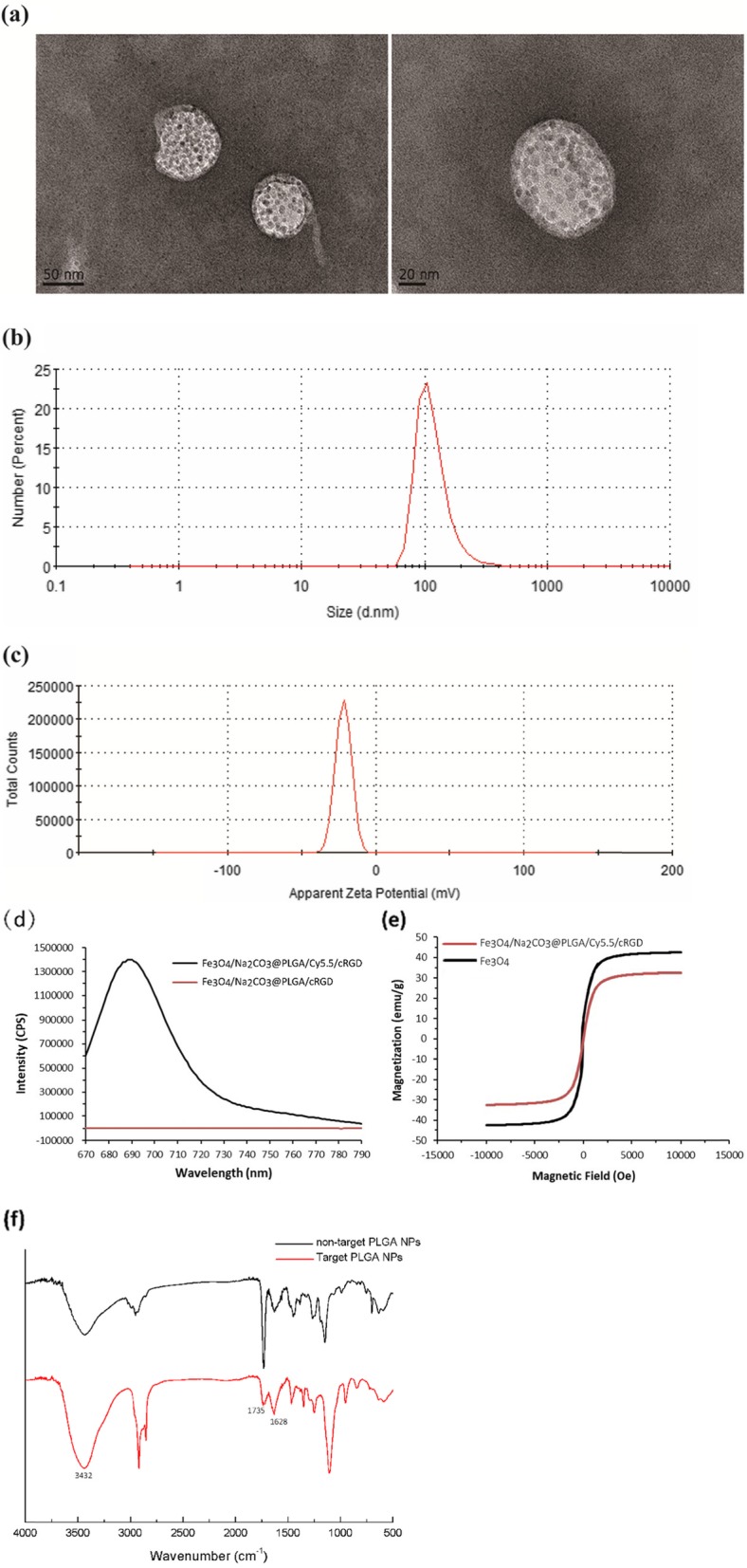
TEM images (**a**) Size distributions (**b**) Zeta potential (**c**) of Fe_3_O_4_/Na_2_CO_3_@PLGA/Cy5.5/cRGD NPs. **d** Fluorescence emission spectrum of Fe_3_O_4_/Na_2_CO_3_@PLGA/Cy5.5/cRGD and Fe_3_O_4_/Na_2_CO_3_@PLGA/cRGD NPs. **e** Magnetic hysteresis curves of Fe_3_O_4_/Na_2_CO_3_@PLGA/Cy5.5/cRGD NPs and Fe_3_O_4_ NPs. **f** The FTIR of spectra of targeted Fe_3_O_4_/Na_2_CO_3_@PLGA/Cy5.5/cRGD and non-targeted PLGA NPs

Transmission electron microscopy images showed that Na_2_CO_3_/Fe_3_O_4_@PLGA/Cy5.5/RGD NPs were clear spheres with uniformly dispersed iron oxide particles visible in the shell (Fig. 1a). The average hydrodynamic size of the NPs was measured as 117.6 nm by dynamic light scattering, and the average polydispersity index was 0.234 (Fig. 1b). The surface charge of the NPs was confirmed by zeta potential measurements to be − 21.7 mV (Fig. 1c). Fluorescence spectrum measurement revealed that Na_2_CO_3_/Fe_3_O_4_@PLGA/Cy5.5/RGD had the maximum emission wavelength at 685 nm, indicating that Cy5.5 was successfully encapsulated in the PLGA core (Fig. 1d). The saturation magnetization values for Na_2_CO_3_/Fe_3_O_4_@PLGA/Cy5.5/RGD and free Fe_3_O_4_ NPs were equal to 32.6 and 42.5 emu/g, respectively (Fig. 1e). These findings indicated the superparamagnetic characteristic of the nanoparticles at room temperature. The FITR spectrum of Na_2_CO_3_/Fe_3_O_4_@PLGA/Cy5.5/RGD showed that N-H stretching vibration and –OH absorption peak appeared around 3432 cm^−1^. In addition, we found an enhancement (1628 cm^−1^) of the C = O stretching vibration. Compared to that of the non-targeted NPs, the characteristic peak (the carboxyl) at 1735 cm^−1^ of the targeted NPs was significantly reduced. The results showed the bonding between the carboxyl group on the microsphere surface and the amino group on RGD peptide. In vitro binding of Na_2_CO_3_/Fe_3_O_4_@PLGA/Cy5.5/RGD NPs is shown in Fig. 1f.

### In vitro binding of Na2CO3/Fe3O4@PLGA/Cy5.5/RGD NPs

**Fig. 2:**
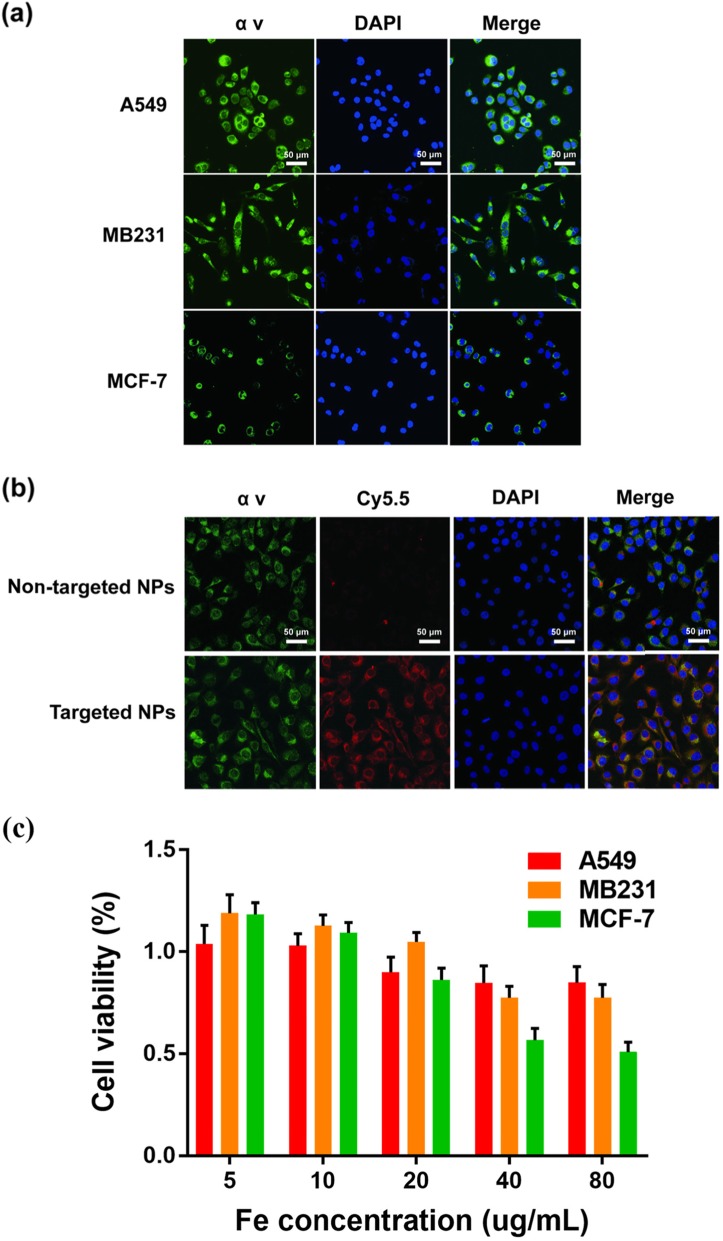
**a** Confocal fluorescence images with the expression of αv integrin on MB231, A549, and Mcf-7 cells. Blue and green represent DAPI and αv fluorescence, respectively. **b** Confocal fluorescence images of MB231 cells incubated with targeted Fe_3_O_4_/Na_2_CO_3_@PLGA/Cy5.5/cRGD NPs and non-targeted NPs. Blue, red, and green, represent DAPI, Cy5.5, and α v fluorescence, respectively. **c** Relative viability of MB231 cells incubated with different concentrations of Fe_3_O_4_/Na_2_CO_3_ @PLGA/Cy5.5/cRGD NPs

αvβ3 integrin is generally highly expressed on breast cancer tumour endothelial cells and can promote tumour metastasis [33–36]. Cellular immunofluorescence for the expression of αv integrin in MDA-MB-231 cells was much higher than that in MCF-7 cells; A549 cells served as positive controls (Fig. 2a). The cellular uptake of NPs was studied by CLSM (Fig. 2b). The Na_2_CO_3_/Fe_3_O_4_@PLGA/Cy5.5/RGD NPs showed a much higher binding rate to MD-MB-231 cells than did non-targeted NPs. The fluorescence images also revealed that Na_2_CO_3_/Fe_3_O_4_@PLGA/Cy5.5/RGD NPs bound to the cell cytoplasm, and merged images showed the same locations as the expression of αv integrin [37, 38].

### Cytotoxicity assay

The in vitro cytotoxicity of Na_2_CO_3_/Fe_3_O_4_@PLGA/Cy5.5/RGD NPs was estimated in MDA-MB-231 cells using a CCK8 assay, while A549 and MCF-7 cells treated with NPs were used as controls (Fig. 2c). In the range of Fe concentrations of 5–80 μg/mL, the cell viability of A549 and MB231 cells was not significantly reduced, and both were above 70%. In contrast, MCF-7 cells showed a significant decrease in cell viability to approximately 50% at Fe concentrations higher than 40 μg/mL. The CCK8 results demonstrated that Na_2_CO_3_/Fe_3_O_4_@PLGA/Cy5.5/RGD NPs showed significantly lower cytotoxicity in MDA-MB-231 cells over a given concentration range.

### In vitro contrast imaging

**Fig. 3:**
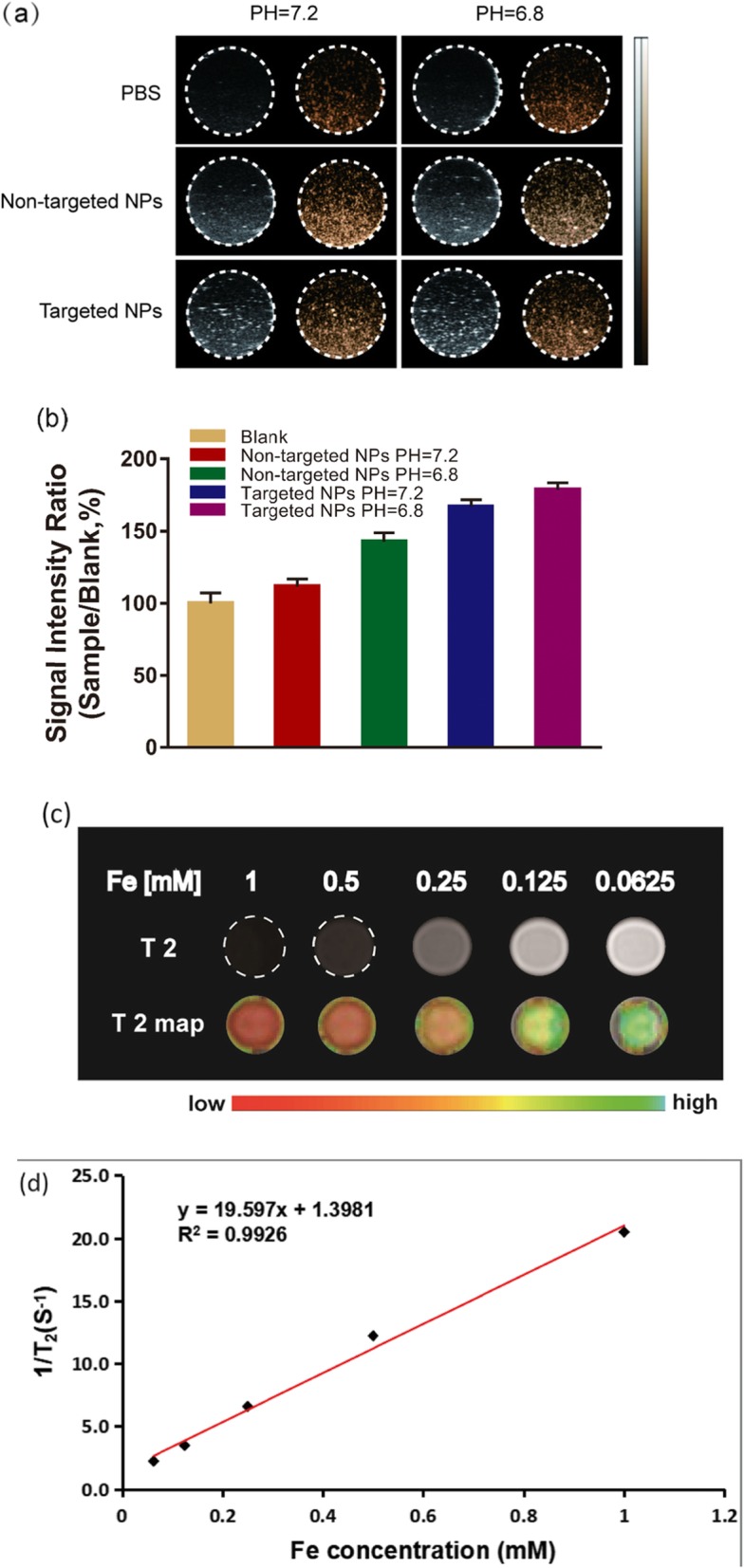
**a** Ultrasonic images of the targeted NPs and non-targeted NPs recorded at different pH values (7.2 and 6.8), PBS as a control. **b** Signal intensity rate is calculated by sample/blank, sample represents the echo intensity of the targeted and non-targeted NPs, and blank represents the echo intensity of PBS. **c** T2-weighted MR images of Fe_3_O_4_/Na_2_CO_3_@PLGA/Cy5.5/cRGD NPs with different Fe concentrations (0.0625, 0.125, 0.25, 0.5, and 1 mM). **d** The transverse relativities (r2) were 19.597 mM^−1^ s^−1^ for Fe_3_O_4_/Na_2_CO_3_@PLGA/Cy5.5/cRGD NPs

We used an agar gel phantom to study the performance of Na_2_CO_3_/Fe_3_O_4_@PLGA/Cy5.5/RGD NPs in vitro at different pH values (Fig. 3a). The ultrasound contrast images of Na_2_CO_3_/Fe_3_O_4_@PLGA/Cy5.5/RGD NPs were significantly enhanced at weakly acidic pH (pH 6.8) compared with pH 7.2, likely because pH 7.2 does not produce enough CO_2_ bubbles for ultrasound imaging. In contrast, when the NPs were in a weakly acidic environment, enough bubbles could be generated for ultrasound imaging. This characteristic is relevant to tumours, which exhibit high tissue heterogeneity and diverse pH levels (pH 6.8–7.2) in vivo [32, 39, 40]. The signal intensity of the ultrasound image was then analyzed (Fig. 3b). The signal intensity ratios of the non-targeted NP (pH = 7), non-targeted NP (PH = 5), targeted NP (PH = 7), and targeted NP (PH = 5) groups relative to the signal intensity of the blank group were 112%, 145%, 167%, and 178 ± 4%, respectively, which clearly indicates that the targeted NP group (PH = 5) had the strongest US signal.

For the MRI study in vitro, as the concentration of Fe in the Na_2_CO_3_/Fe_3_O_4_@PLGA/Cy5.5/RGD NPs increased, the T2-weighted signal intensity showed a significant decrease, indicating the possibility of these NPs for use as T2 MR contrast agents (Fig. 3c). The transverse relaxation rate (r2) of Na_2_CO_3_/Fe_3_O_4_@PLGA/Cy5.5/RGD NPs was calculated to be 19.597 mM^−1^ s^−1^. Although the transverse relaxation rate (r2) of Na_2_CO_3_/Fe_3_O_4_@PLGA/Cy5.5/RGD NPs is lower than that of many other MRI superparamagnetic agents, the composition of Fe_3_O_4_ can increase the r2, which was 2.94 times higher than the r2 of SPIO particles used clinically.

### Ultrasound contrast imaging of Na_2_CO_3_/Fe_3_O_4_@PLGA/Cy5.5/RGD NPs

**Fig. 4:**
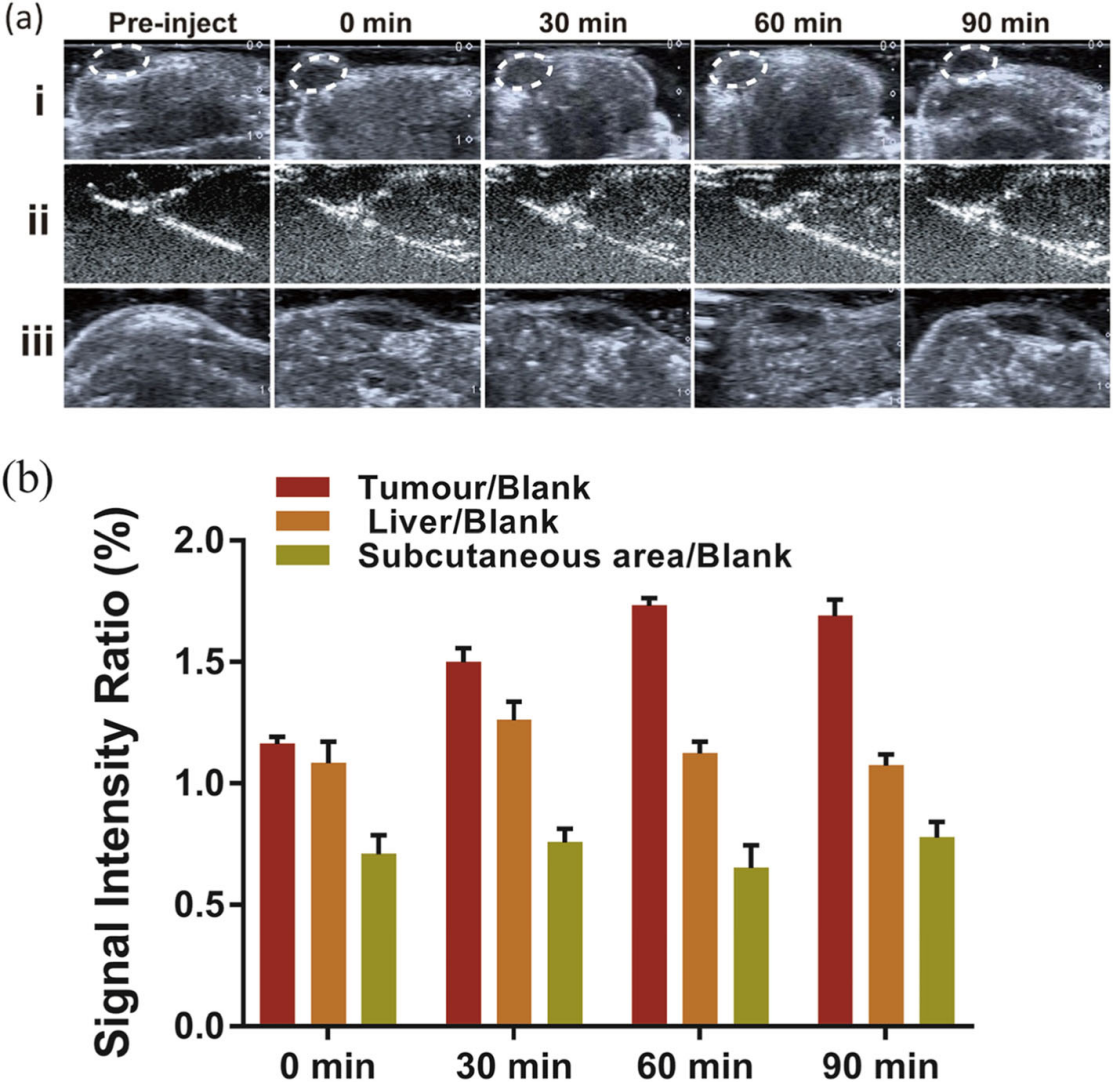
**a** In vivo ultrasound imaging of tumors, livers, and subcutaneous areas at different time after injection of Fe_3_O_4_/Na_2_CO_3_@PLGA/Cy5.5/cRGD NPs. **b** Echo intensity rate as a function of time is calculated by tissue/blank, tissue represents the echo intensity of the tumor, liver, or subcutaneous area, blank represents the echo intensity before injection

To demonstrate the potential of the Na_2_CO_3_/Fe_3_O_4_@PLGA/Cy5.5/RGD NPs for ultrasound imaging in tumours, we administered a tail vein injection of Na_2_CO_3_/Fe_3_O_4_@PLGA/Cy5.5/RGD NPs to breast cancer xenograft nude mice and monitored the ultrasound images as a function of time (Fig. 4a). Before injection, images of the tumour, liver, and subcutaneous area were recorded. Immediately after injection, the area of the tumour tissues did not show any contrast enhancement. Enhancement of the tumour area was observed beginning 30 min after injection and lasted for 90 min. The in vivo ultrasound results showed that Na_2_CO_3_/Fe_3_O_4_@PLGA/Cy5.5/ RGD NPs generated enough bubbles in acidic tumour tissues to produce echogenic reflectivity for ultrasound imaging. As a control, we also obtained images of the liver and subcutaneous tissues at different times after injection of the targeted NPs. Throughout the observation period, no significant enhancement was found in the subcutaneous injection area, and the enhancement in the liver, which decreased over time, was significantly lower than that in the tumours (Fig. 4b). This result indicates that Na_2_CO_3_/Fe_3_O_4_@PLGA/Cy5.5/RGD NPs circulating in the body at physiological pH do not produce a substantial amount of CO_2_ bubbles for ultrasound contrast enhancement.

### MRI of Na_2_CO_3_/Fe_3_O_4_@PLGA/Cy5.5/RGD NPs

**Fig. 5:**
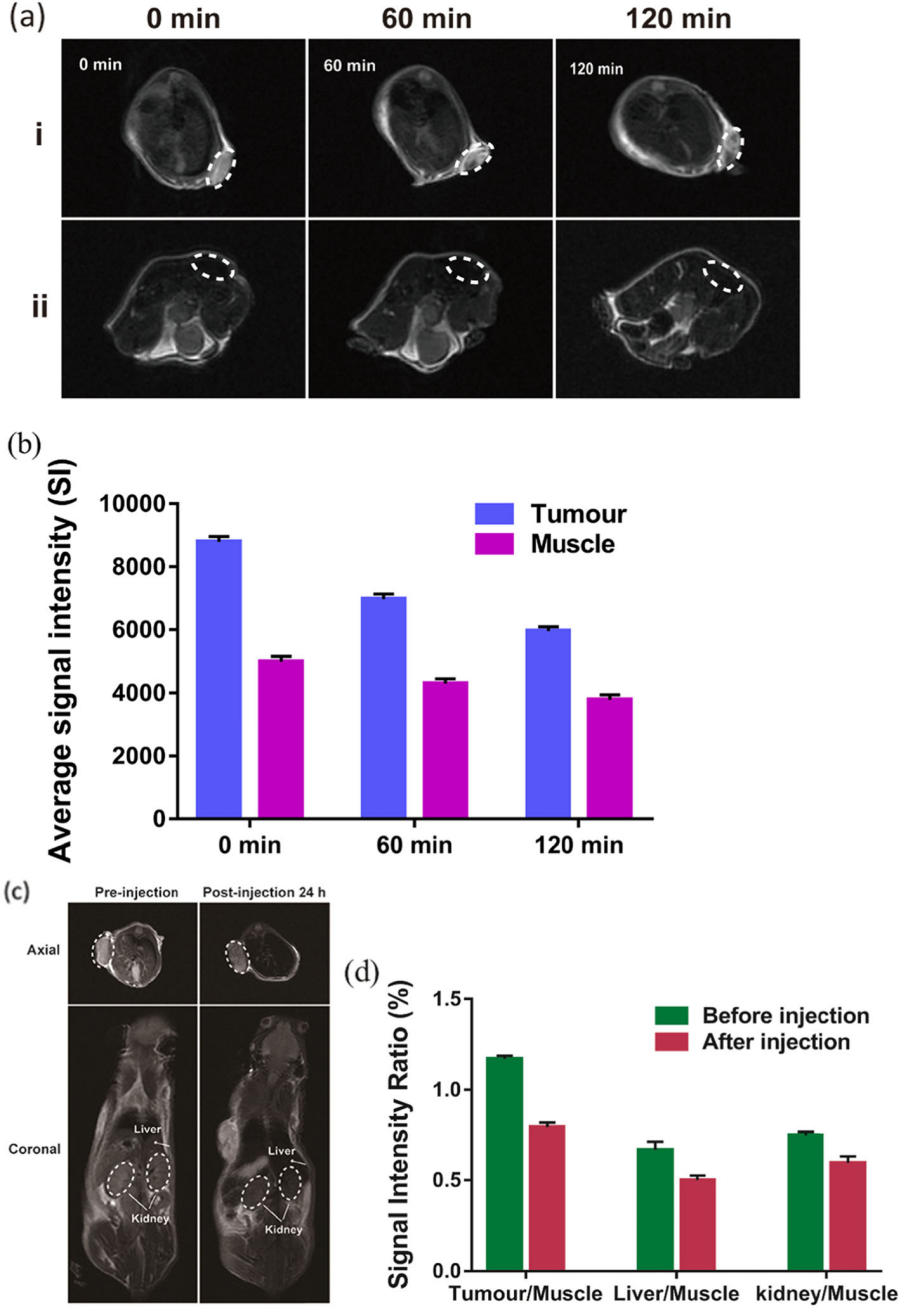
**a** In vivo T2-MR images of normal and tumor subcutaneous tissues before and after injection of Fe_3_O_4_/Na_2_CO_3_@PLGA/Cy5.5/cRGD NPs. **b** Average signal intensity in muscle and tumor for injection of Fe_3_O_4_/Na_2_CO_3_@PLGA/Cy5.5/cRGD NPs. **c** T2-MR images on axial and coronal of MDA-MB-231 tumor-bearing mice before and after intravenous injection of Fe_3_O_4_/Na_2_CO_3_@PLGA/Cy5.5/cRGD NPs. **d** Signal intensity ratio is calculated by tissue/muscle, tissue stands for the signal intensity of the tumor, liver, and kidney before and after injection of the targeted NPs, muscle stands for the signal intensity of the muscle at the same time

For in vivo MRI, to demonstrate that NPs can be used for tumour-specific imaging, Na_2_CO_3_/Fe_3_O_4_@PLGA/Cy5.5/RGD NPs were injected directly into tumours and muscles. The results showed that the tumour area exhibited a significant decrease in T2-MR contrast after injection of the targeted NPs, and the signal intensity decreased significantly from 8875 at 0 min to 5972 at 120 min after injection (Fig. 5a, b). However, with the same amount of nanoparticles injected, the subcutaneous muscle area showed a much lower T2 signal decrease. This finding demonstrates the effectiveness of Na_2_CO_3_/Fe_3_O_4_@PLGA/Cy5.5/RGD NPs with hypersensitive integrin-targeted T2-MR contrast agents for use in tumour-targeted imaging. In the tail vein injection group, T2-MR imaging also showed an obvious decrease in contrast in the tumour at 24 h post-injection, demonstrating the high tumour accumulation of Na_2_CO_3_/Fe_3_O_4_@PLGA/Cy5.5/RGD NPs (Fig. 5c, d). Moreover, decreased T2 signals were observed in the liver and kidneys, indicating that the iron ions in the NPs could be rapidly cleared from the body. Therefore, MRI revealed that the PLGA-wrapped Fe_3_O_4_ nanoparticles exhibited efficient passive tumour targeting via the enhanced permeability and retention (EPR) effect, especially RGD-mediated targeting, but could be decomposed and quickly excreted in vivo.

### Fluorescence imaging and histology

**Fig. 6:**
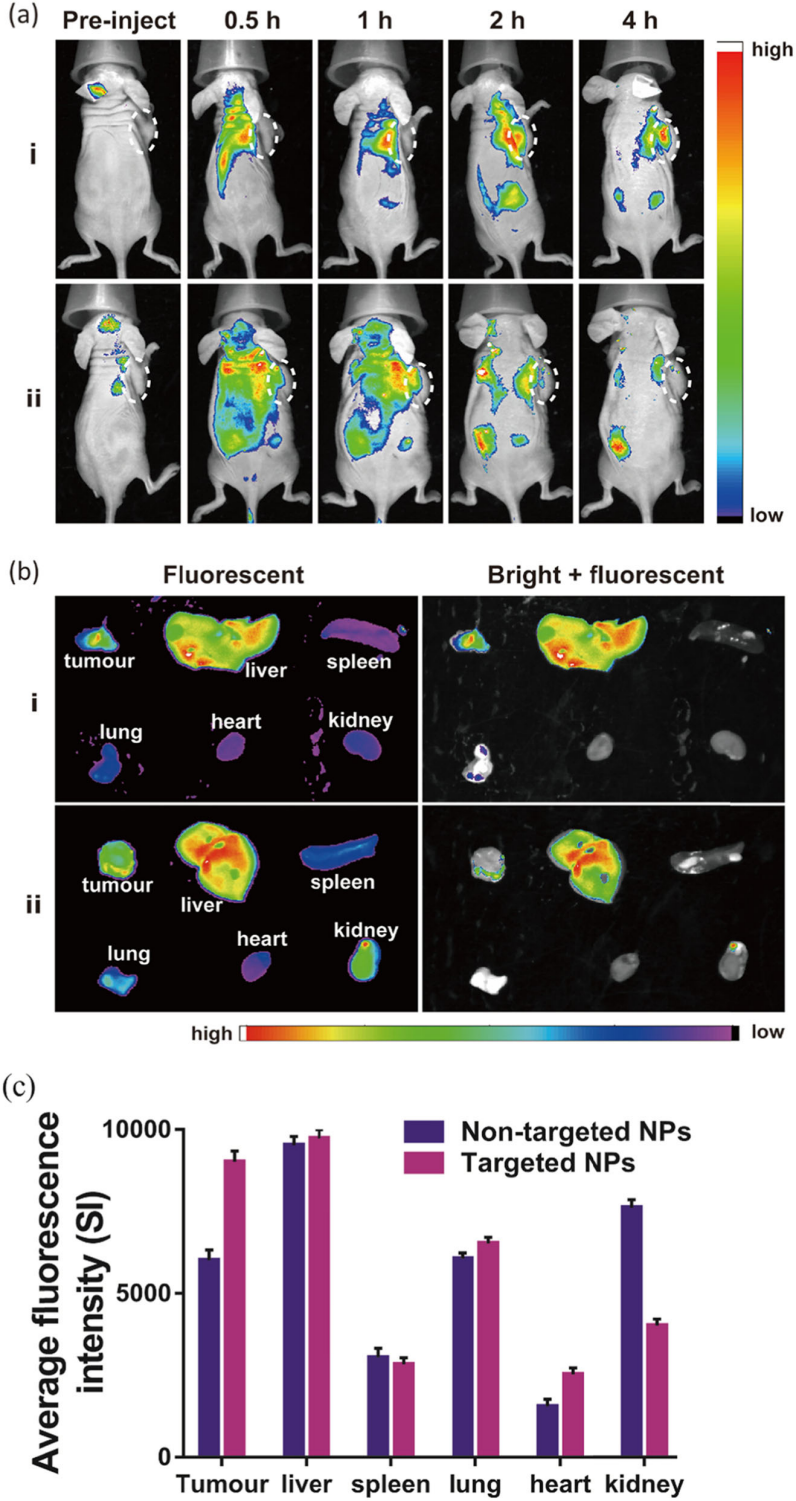
**a** In vivo fluorescence fluorescence imaging of animals at 0, 0.5, 1, 2, and 3 h post-injection after injection of targeted and non-targeted NPs. b Ex vivo fluorescence images of tumors and major organs (liver, spleen, lung, heart, and kidney) collected from animals. **c** Averaged fluorescence intensity of various organs and tumors

Two hundred microliters of NPs were intravenously injected into mice for in vivo fluorescence imaging. In the group injected with RGD-targeted NPs, the fluorescence signal of Cy5.5 gradually increased in the tumour area and reached a peak at 4 h after injection, indicating that Na_2_CO_3_/Fe_3_O_4_@PLGA/Cy5.5/RGD NPs can effectively accumulate in the tumour. In the non-targeted group, NPs were distributed throughout the body after injection and rapidly cleared, and they did not accumulate in the tumour for a long period of time (Fig. 6a). The mice were then dissected, and the main organs and tumours were collected for in vitro fluorescence imaging, which revealed high tumour uptake of the targeted NPs (Fig. 6b, c). The fluorescence intensity of Cy5.5 in the tumours of mice injected with targeted NPs was 1.5 times that in mice injected with non-targeted NPs.

**Fig. 7:**
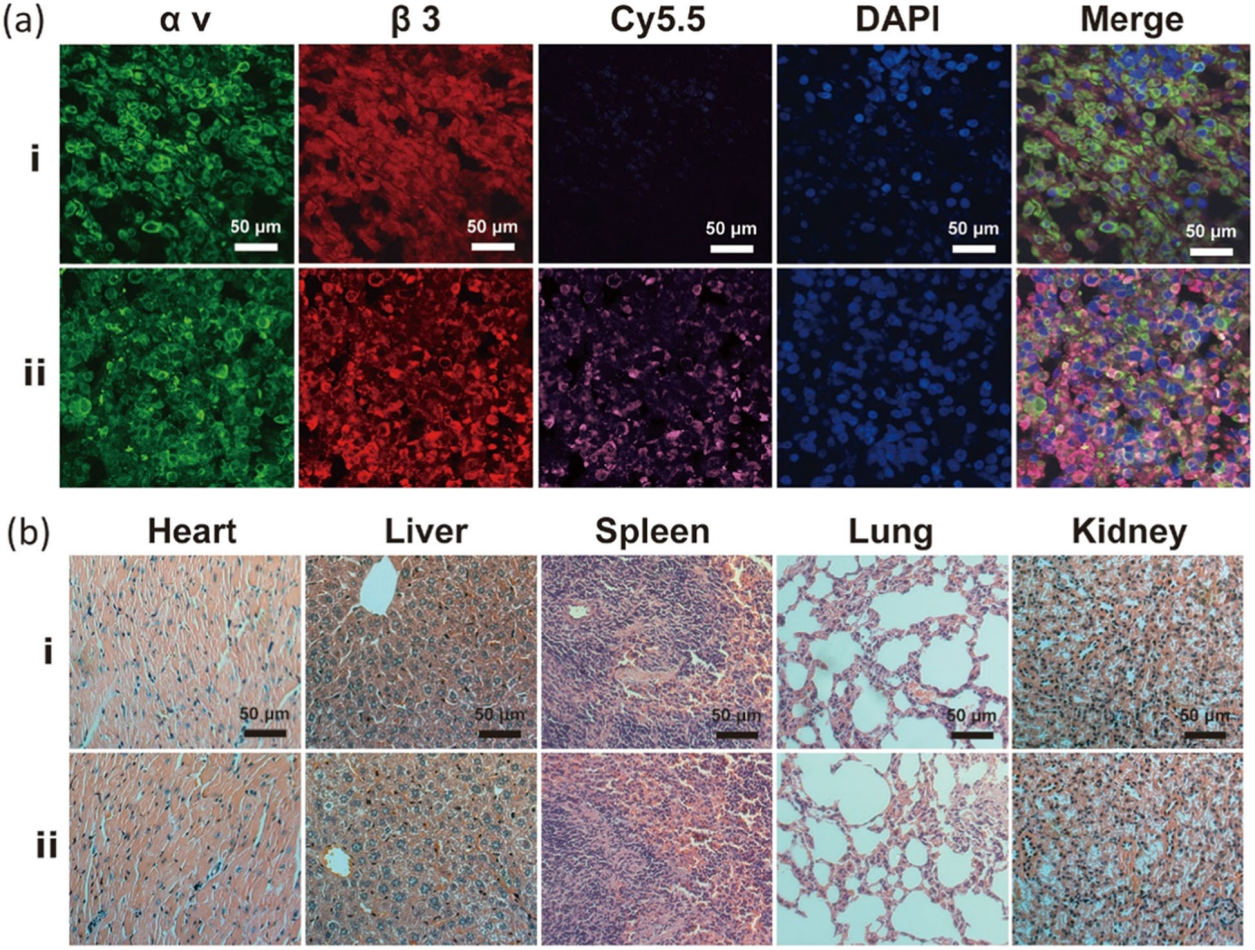
**a** Fluorescent imaging of MDA-MB-231 tumor frozen sections from mice injected with targeted and non-targeted NPs. Green, red, purple, and blue represent αv, β3, Cy5.5, and DAPI fluorescence, respectively. **b** H&E-stained tumor slices collected from mice after injection of targeted and non-targeted NPs

Furthermore, tumour-specific targeting of Na_2_CO_3_/Fe_3_O_4_@PLGA/Cy5.5/RGD NPs was verified by tissue fluorescence imaging of frozen tumour sections (Fig. 7a). Immunofluorescence staining of tumour sections with antibodies against αv and β3 integrin revealed significant expression of αvβ3 integrin in tumour tissues. The fluorescence of αv and β3 integrin was merged with the Cy5.5 fluorescence of Na_2_CO_3_/Fe_3_O_4_@PLGA/Cy5.5/RGD NPs to obtain an immunostaining image revealing colocalization. The immunofluorescence results in the tumour tissues indicated that Na_2_CO_3_/Fe_3_O_4_@PLGA/Cy5.5/RGD NPs specifically bind to αvβ3 integrin in MB231 malignant breast cancer. Moreover, H&E staining of Na_2_CO_3_/Fe_3_O_4_@PLGA/Cy5.5/RGD NPs compared with the non-targeted group showed that all organ tissue sections had normal pathological morphology and no histopathological damage response (Fig. 7b). All the results of the above cytotoxicity and histological analysis indicated that Na_2_CO_3_/Fe_3_O_4_@PLGA/Cy5.5/RGD NPs induced no significant toxicity to major organ tissues in vivo, and their good biocompatibility can be reasonably attributed to PLGA.

## Conclusions

In conclusion, the results above demonstrate a creative and successful approach for MRI of breast cancer through a magnetic targeting and a gas-generating system that is activated in the tumour microenvironment. Na_2_CO_3_/Fe_3_O_4_@PLGA/Cy5.5/RGD NPs exhibit excellent imaging performance and good biocompatibility in MR/ultrasound/fluorescent imaging modes. Our work shows the strong potential for tumour diagnosis with improved multi-modal imaging.

## Materials and methods

### Materials

Poly (lactic-co-glycolic acid) (PLGA) (lactide:glycolide = 75:25, Mw = 20,000), Cyanine5.5 dye, and polyvinyl alcohol (PVA) were purchased from Sigma-Aldrich Company (Shanghai, China). The RGD peptide was custom synthesized by GenicBio BioTech Co. Ltd. (Shanghai, China). Fe_3_O_4_ nanoparticles and sodium carbonate (Na_2_CO_3_) were purchased from Xian Ruixi Biological Technology Co. Ltd. (Henan, China). Dichloromethane (CH_2_Cl_2_) and dimethyl sulfoxide (DMSO) were obtained from Solarbio Company (Beijing, China). All chemicals were of analytical grade.

### Synthesis of Fe3O4/Na2CO3@PLGA/Cy5.5/cRGD nanoparticles

First, 12.5 mg of PLGA and 0.25 mL of chloroform were mixed together. Then, 5 μL of Cy5.5, 15 μL of oleic acid-modified magnetic nanoparticles dispersed in chloroform (OA@Fe_3_O_4_, 10 mg/mL), 5 μL of sodium carbonate (Na_2_CO_3_), and 1.5 mL of 1% PVA solution were added in sequence and emulsified with an ultrasonic processor for 2 min. Then, 12.5 mL of 0.3% PVA solution was added and stirred for 3–4 h at room temperature, and 12.5 mL of 0.4% PVA solution was added for stirring (500 rpm/min) overnight to remove residual organic solvent. The above solution was subjected to several ultrafiltration washings with ddH_2_O and then diluted with PB buffer (pH = 7.4) to a final volume of 1.25 mL. Next, 0.25 mg of EDC and 1.25 mg of NHS were added to the above mixed solution. The mixture was stirred for 30 min at 25 °C and then, washed three times with ultrafiltration and resuspended in PB buffer solution (pH = 7.4). Next, 1.25 mg of cRGD was added to the solution and stirred at 4 °C overnight. To remove EDC, NHS, and any residual cRGD, the transparent solution was filtered by an ultrafiltration tube. Finally, Fe_3_O_4_/Na_2_CO_3_@PLGA/Cy5.5/cRGD NPs were resuspended in 1.25 mL of deionized water and stored at 4 °C.

### Nanoparticle characterization

The dynamic diameters and zeta potential of nanoparticles were measured by a Zetasizer Nano-ZS (Malvern Instruments, UK). The morphology of the nanoparticles was obtained using a FEI Tecnai F20 transmission electron microscope. The Cy5.5 loading was recorded by a Hitachi F-7000 fluorescence spectrometer. FTIR was performed using a fourier transform infrared spectrometer (Alpha II, Bruker, Switzerland). A vibrating sample magnetometer (VSM, Lake Shore 7410) was applied to determine the hysteresis curve of the nanoparticles and free Fe_3_O_4_.

### Cells and animals

Human breast cancer MDA-MB-231 cells were kindly provided by the Stem Cell Bank, Chinese Academy of Sciences. Cells were maintained at 37 °C in 95% air and 5% CO_2_. Female BALB/c mice (4 weeks) were purchased from Shanghai Slaccas Laboratory Animal Co. Ltd. and maintained according to protocols approved by the Guangxi Medical University Laboratory Animal Center. Animal experiments were followed with the Guide for Care and Use of Laboratory Animals, released by the Animals Ethics Committee of Laboratory Animal Center, Guangxi Medical University. MDA-MB-231 breast cancer cells were transplanted into the right flank of BALB/c mice (2 × 106 in 200 μl cells per mouse) and allowed to grow for 10–14 days (mean diameter 5 mm) before imaging.

### Cellular expression of αv integrin

Cellular immunofluorescence was carried out to confirm the high expression of αv integrin in MDA-MB-231 cells. A549 and MCF-7 cells were used as controls. Cells were seeded onto 35 mm glass-bottom culture dishes (MatTek, USA) at 2 × 10^4^ cells mL^−1^ for 24 h. After incubation, cells were fixed for 20 min at room temperature with 4% paraformaldehyde. Then, they were incubated with rabbit monoclonal anti-integrin αv antibody (ab179475, Abcam) at 4 °C overnight and anti-rabbit IgG antibody for 1 h at room temperature. Finally, the cells were stained with DAPI. Images were acquired by confocal laser scanning microscopy (TCS SP8, Leica, Germany).

To assess the targeting efficacy of the nanoparticles, a cellular uptake study was performed by using confocal laser scanning microscopy (CLSM). Cells were seeded onto 35 mm glass-bottom culture dishes (MatTek, USA) at 2 × 10^4^ cells mL^−1^ for 24 h. Then, the cells were incubated with RGD-targeted NPs (30 μg mL^−1^, 0.5 mL) at pH 7.4 for 2 h, and non-targeted NPs were used as controls. After incubation, the cells were fixed with 4% paraformaldehyde for 20 min and then incubated with αv antibody. By means of colocalization, we verified the targeted binding of the nanoparticles to the integrin on the cells.

### CCK8 assay

The biocompatibility of Na_2_CO_3_/Fe_3_O_4_@PLGA/Cy5.5/RGD nanoparticles was evaluated by a cytotoxicity study. MDA-MB-231, A549, and MCF-7 cells were seeded onto 96-well plates at 5 × 10^3^ cells mL^−1^ for 24 h. Then, 0.1 mL of Na_2_CO_3_/Fe_3_O_4_@PLGA/Cy5.5/RGD NP suspension at Fe concentrations of 5, 10, 20, 40, and 80 μg/mL was added to each well and incubated for 24 h. Finally, 10 μL of CCK8 solution was added, and the suspension was incubated for another 1 h. The results were determined by a microplate reader (Thermo Scientific, USA) at 450 nm.

### Contrast-enhanced ultrasound imaging

Ultrasound imaging of nanoparticles was performed by using a Vevo 2100 (Fujifilm Visual Sonics Inc., Canada) ultrasound system. The RGD-targeted and non-targeted NPs were added to the agarose model, and PBS was used as a control. Images were recorded in B-mode and CEUS-mode with different pH buffers of 7.2 and 6.8. The area of interest was drawn, and the average grey value was measured in B-mode images.

For in vivo ultrasound imaging, mice were anaesthetized with 2% isoflurane (Hebei Yipin Pharmaceutical Co., Ltd., China), and body temperature was maintained at 37 °C with a heated pad. A total of 200 μL of RGD-targeted NPs was injected via the tail vein. Control animals received a subcutaneous injection with the same amount of NPs. Ultrasonic images were recorded using a 7-MHz transducer to continuously acquire ultrasound images of the tumours, livers, and subcutaneous areas. The acoustic focus zone was placed at the centre of the tumour with the largest cross-section, and a field of view containing the tumour and its adjacent tissue was obtained.

### Magnetic resonance imaging

MRI exams of Na_2_CO_3_/Fe_3_O_4_@PLGA/Cy5.5/RGD NPs were performed using a 3.0 T MR (GE Healthcare, USA) and an animal coil (RF TECH LIMITED, China). NPs with different Fe concentrations of 0.031, 0.063, 0.125, 0.25, 0.5, and 1 mM were scanned in 1 mL Eppendorf tubes, and PBS was used as a control. T2 MRI was performed for each tube using a T2-weighted FSE sequence (slice thickness of 3 mm, TR/TE 2000/74.4 ms, 8 × 8 cm FOV, and 320 × 256 matrix). The relaxivities (r2) were calculated by a linear fitting of the inverse relaxation time as a function of the Fe concentration.

For in vivo MRI, mice were randomly divided into two groups (*n* = 3) for MR scanning that received either (1) local injection of Na_2_CO_3_/Fe_3_O_4_@PLGA/Cy5.5/RGD NPs within subcutaneous muscle and tumour tissues or (2) tail vein injection of Na_2_CO_3_/Fe_3_O_4_@PLGA/Cy5.5/RGD NPs. Baseline images of the mice were taken before injection of the nanoparticles. For group one, the same amount of NPs was injected into the subcutaneous and tumour tissues, and MRI scanning was performed every 30 min to record the signal transition of the tissues. For group two, tumour imaging was performed in the axial and coronal positions, and the MR parameters were the same as those used for in vitro imaging. The signal intensity (SI) in the region of interest (ROI) was measured and compared to tissue signals at different times before and after injection.

### Tumour fluorescence imaging

For fluorescence imaging in vivo, an in vivo fluorescence imaging system (FX PRO, Bruker, Switzerland) was used for scanning, and the mice were randomly divided into two groups (*n* = 3): (1) RGD-targeted NPs and (2) non-targeted NPs. Images were captured every 30 min for a period of 4 h after the injection. Subsequently, important organs and tumours were harvested and imaged, and the distribution of fluorescence in the organs of the body was observed. Quantitative analysis of fluorescence intensity was performed using molecular imaging software (Bruker, Switzerland). These important organs then underwent H&E staining to evaluate the tissue toxicity. Frozen tumour sections were also subjected to fluorescence immunostaining with antibodies against αv integrin and β3 integrin.

## Data Availability

The conclusions made in this manuscript are based on the data which are all presented and shown in this paper.

## Abbreviations

Cy5.5: Sulfo-Cyanine5.5 NHS ester
DAPI: 4′,6-diamidino-2-phenylindole
EDC: 1-Ethyl-3-(3-dimethylaminopropyl) carbodiimide hydrochloride
FSE: Fast spin echo
MR: Magnetic resonance
NHS: N-hydroxysuccinimide
PB: Phosphate buffer
PBS: Phosphate buffered saline
PEG: Polyethylene glycol
PVA: Polyvinyl alcohol
RGD: Arginine–glycine–aspartate
TE: Echo time
TR: Repetition time

## References

[1] Jai Il P, Dinesh J, Ross W (2010). Microbubbles loaded with nanoparticles: a route to multiple imaging modalities. Acs Nano..

[2] Fabian K, Stanley F, Patrick K, Wiltrud L, Twan L (2012). Ultrasound microbubbles for molecular diagnosis, therapy, and theranostics. J Nuclear Med.

[3] Mcewan C, Owen J, Stride E (2015). Oxygen carrying microbubbles for enhanced sonodynamic therapy of hypoxic tumours. J Controlled Release..

[4] Min HS, Kang E, Koo H (2012). Gas-generating polymeric microspheres for long-term and continuous in vivo ultrasound imaging. Biomaterials.

[5] Bloch SH, Wan M, Dayton PA, Ferrara KW (2004). Optical observation of lipid- and polymer-shelled ultrasound microbubble contrast agents. Appl Physics Letters.

[6] Otani K, Nishimura H, Kamiya A, Harada-Shiba M (2018). Simplified preparation of α v β 3 integrin-targeted microbubbles based on a clinically available ultrasound contrast agent: validation in a tumor-bearing mouse model. Ultrasound Med Biol.

[7] Wilson SR, Burns PN (2010). Microbubble-enhanced US in body imaging: what role?. Radiology..

[8] Yang H, Cai W, Xu L et al (2015) Nanobubble–Affibody: novel ultrasound contrast agents for targeted molecular ultrasound imaging of tumor. Biomaterials. 37:279–288 10.1016/j.biomaterials.2014.10.013 25453958

[9] Li Y, An H, Wang X (2018). Ultrasound-triggered release of sinoporphyrin sodium from liposome-microbubble complexes and its enhanced sonodynamic toxicity in breast cancer. Nano Research..

[10] Yu L, Hu P, Chen Y. (2018) Gas-Generating Nanoplatforms: Material Chemistry, Multifunctionality, and Gas Therapy. Adv Materials. 30(49):e1801964 10.1002/adma.201801964 30066474

[11] Kun Z, Huixiong X, Hangrong C et al (2015) CO2 bubbling-based ‘nanobomb’ system for targetedly suppressing Panc-1 pancreatic Tumor via low intensity ultrasound-activated inertial cavitation. Theranostics. 5(11):1291–1302 10.7150/thno.12691 PMC4568455 26379793

[12] Kyung Hyun M, Hyun Su M, Jae LH (2015). pH-controlled gas-generating mineralized nanoparticles: a theranostic agent for ultrasound imaging and therapy of cancers. Acs Nano..

[13] Min HS, Son S, Dong GY (2016). Chemical gas-generating nanoparticles for tumor-targeted ultrasound imaging and ultrasound-triggered drug delivery. Biomaterials..

[14] Ke CJ, Chiang WL, Liao ZX (2013). Real-time visualization of pH-responsive PLGA hollow particles containing a gas-generating agent targeted for acidic organelles for overcoming multi-drug resistance ☆. Biomaterials..

[15] Kircher MF, Gambhir SS, Jan G (2011). Noninvasive cell-tracking methods. Nat Reviews Clin Oncol.

[16] Kircher MF, Willmann JK (2012). Molecular body imaging: MR imaging, CT, and US. part I. principles. Radiology..

[17] Wang P, Yong F, Lu L (2018). NIR-II nanoprobes in-vivo assembly to improve image-guided surgery for metastatic ovarian cancer. Nature Comm.

[18] Teng L, Sixiang S, Chao L (2015). Iron oxide decorated MoS2 nanosheets with double PEGylation for chelator-free radiolabeling and multimodal imaging guided photothermal therapy. Acs Nano..

[19] Cai W, Gao H, Chu C (2017). Engineering phototheranostic nanoscale metal-organic frameworks for multimodal imaging-guided cancer therapy. Acs Appl Mater Interfaces..

[20] Atabaev TS (2018). PEG-coated superparamagnetic dysprosium-doped Fe3O4 nanoparticles for potential MRI imaging. BioNanoScience..

[21] Thapa B, Diaz-Diestra D, Beltran-Huarac J, Weiner BR, Morell G (2017). Enhanced MRI T2 relaxivity in contrast-probed anchor-free PEGylated iron oxide nanoparticles. Nanoscale Res Letters..

[22] Li M, Bu W, Jie R (2018). Enhanced synergism of thermo-chemotherapy for liver cancer with magnetothermally responsive nanocarriers. Theranostics..

[23] Bai J, Wang JT, Rubio N (2016). Triple-modal imaging of magnetically-targeted nanocapsules in solid tumours In Vivo. Theranostics..

[24] Jin X, Xu C, Kohler N, Hou Y, Sun S (2010). Controlled PEGylation of monodisperse Fe3O4 nanoparticles for reduced non-specific uptake by macrophage cells. Adv Materials.

[25] Yang J, Luo Y, Xu Y (2015). Conjugation of iron oxide nanoparticles with RGD-modified dendrimers for targeted tumor MR imaging. Acs Appl Materials Interfaces.

[26] Jingchao L, Yao H, Wenjie S (2014). Hyaluronic acid-modified hydrothermally synthesized iron oxide nanoparticles for targeted tumor MR imaging. Biomaterials..

[27] Qi J, Chen C, Zhang X (2018). Light-driven transformable optical agent with adaptive functions for boosting cancer surgery outcomes. Nature Communications..

[28] Cheung S, O’Shea DF (2017). Directed self-assembly of fluorescence responsive nanoparticles and their use for real-time surface and cellular imaging. Nature Communications..

[29] Caruana I, Savoldo B, Hoyos V (2015). Heparanase promotes tumor infiltration and antitumor activity of CAR-redirected T lymphocytes. Nature Med.

[30] Chen Q, Xu L, Liang C, Wang C, Peng R, Liu Z (2016). Photothermal therapy with immune-adjuvant nanoparticles together with checkpoint blockade for effective cancer immunotherapy. Nature Communications..

[31] Ko J, Park K, Kim YS (2007). Tumoral acidic extracellular pH targeting of pH-responsive MPEG-poly(β-amino ester) block copolymer micelles for cancer therapy. J Controlled Release.

[32] Heebeom K, Hyejung L, Sojin L (2010). In vivo tumor diagnosis and photodynamic therapy via tumoral pH-responsive polymeric micelles. Chemical Communications..

[33] Wang H, Xu F, Wang Y, Liu X, Jin Q, Ji J (2013). pH-responsive and biodegradable polymeric micelles based on poly(β-amino ester)-graft-phosphorylcholine for doxorubicin delivery. Polymer Chemistry..

[34] Howard LP, Jonathan C, Klibanov AL, Sanjiv K, Lindner JR (2003). Noninvasive assessment of angiogenesis by ultrasound and microbubbles targeted to alpha(v)-integrins. Circulation..

[35] Zhang J, Feng M, Gang N (2018). 68Ga-BBN-RGD PET/CT for GRPR and integrin αvβ3 imaging in patients with breast cancer. Theranostics..

[36] Qing-Hui Z, Ye-Zi Y, Chao W, Yi H, David O (2009). Cyclic RGD-targeting of reversibly stabilized DNA nanoparticles enhances cell uptake and transfection in vitro. J Drug Targeting..

[37] Zhu Y, Zhang J, Meng F (2016). cRGD-functionalized reduction-sensitive shell-sheddable biodegradable micelles mediate enhanced doxorubicin delivery to human glioma xenografts in vivo. J Controlled Release..

[38] Desgrosellier JS, Cheresh DA (2010). Integrins in cancer: biological implications and therapeutic opportunities. Nature Reviews Cancer..

[39] Luo L, Zhong H, Liu S (2017). Intracellular “activated” two-photon photodynamic therapy by fluorescent conveyor and photosensitizer co-encapsulating pH-responsive micelles against breast cancer. Int J Nanomed.

[40] Lau JTF, Pui-Chi L, Xiong-Jie J, Qiong W, Ng DKP (2014). A dual activatable photosensitizer toward targeted photodynamic therapy. J Med Chem.

